# 4,4′-[Propane-1,3-diylbis(nitrilo­methyl­idyne)]dibenzonitrile

**DOI:** 10.1107/S1600536808018680

**Published:** 2008-06-25

**Authors:** Hoong-Kun Fun, Reza Kia, Hadi Kargar

**Affiliations:** a X-ray Crystallography Unit, School of Physics, Universiti Sains Malaysia, 11800 USM, Penang, Malaysia; b Department of Chemistry, School of Science, Payame Noor University (PNU), Ardakan, Yazd, Iran

## Abstract

The mol­ecule of the title Schiff base compound, C_19_H_16_N_4_, has crystallographic twofold rotation symmetry. The imino group is coplanar with the aromatic ring. Within the mol­ecule, the planar units are parallel, but extend in opposite directions from the central methyl­ene bridge. The packing of the mol­ecules is controlled by C—H⋯π inter­actions.

## Related literature

For values of bond lengths, see Allen *et al.* (1987 ▶). For related structures, see: Li *et al.* (2005 ▶); Bomfim *et al.* (2005 ▶); Glidewell *et al.* (2005 ▶, 2006 ▶); Sun *et al.* (2004 ▶); Habibi *et al.* (2007 ▶).
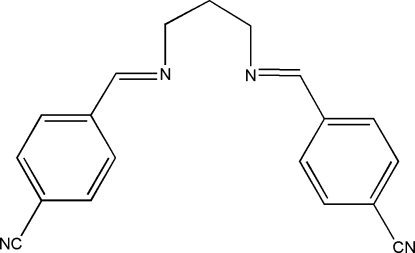


## Experimental

### 

#### Crystal data

C_19_H_16_N_4_
*M* *_r_* = 300.36
Monoclinic, 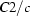

*a* = 14.4982 (4) Å
*b* = 6.9025 (2) Å
*c* = 16.9842 (6) Å
β = 111.659 (4)°
*V* = 1579.67 (8) Å^3^
*Z* = 4
Mo *K*α radiation
μ = 0.08 mm^−1^
*T* = 100.0 (1) K
0.37 × 0.12 × 0.12 mm

#### Data collection

Bruker SMART APEXII CCD area-detector diffractometer
Absorption correction: multi-scan (*SADABS*; Bruker, 2005 ▶) *T*_min_ = 0.886, *T*_max_ = 0.991
13317 measured reflections
1544 independent reflections
1257 reflections with *I* > 2σ(*I*)
*R*_int_ = 0.077

#### Refinement

*R*[*F* ^2^ > 2σ(*F* ^2^)] = 0.081
*wR*(*F* ^2^) = 0.127
*S* = 1.17
1544 reflections
109 parameters
H atoms treated by a mixture of independent and constrained refinement
Δρ_max_ = 0.18 e Å^−3^
Δρ_min_ = −0.37 e Å^−3^

### 

Data collection: *APEX2* (Bruker, 2005 ▶); cell refinement: *APEX2*; data reduction: *SAINT* (Bruker, 2005 ▶); program(s) used to solve structure: *SHELXTL* (Sheldrick, 2008 ▶); program(s) used to refine structure: *SHELXTL*; molecular graphics: *SHELXTL*; software used to prepare material for publication: *SHELXTL* and *PLATON* (Spek, 2003 ▶).

## Supplementary Material

Crystal structure: contains datablocks global, I. DOI: 10.1107/S1600536808018680/tk2276sup1.cif

Structure factors: contains datablocks I. DOI: 10.1107/S1600536808018680/tk2276Isup2.hkl

Additional supplementary materials:  crystallographic information; 3D view; checkCIF report

## Figures and Tables

**Table 1:** Hydrogen-bond geometry (Å, °)

*D*—H⋯*A*	*D*—H	H⋯*A*	*D*⋯*A*	*D*—H⋯*A*
C8—H8*B*⋯*Cg*1^i^	0.97	2.85	3.58	133

Symmetry code: (i) 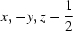. *Cg*1 is the centroid of the C1–C6 benzene ring.

**Fig. 1.**
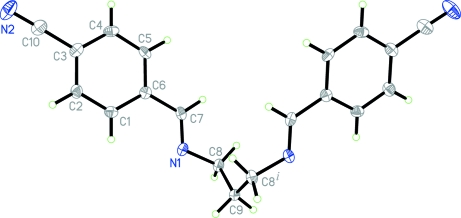
The molecular structure of (I) showing atom labeling and 50% probability ellipsoids [symmetry code for i: -x, y, 0.5 - z].

**Fig. 2.**
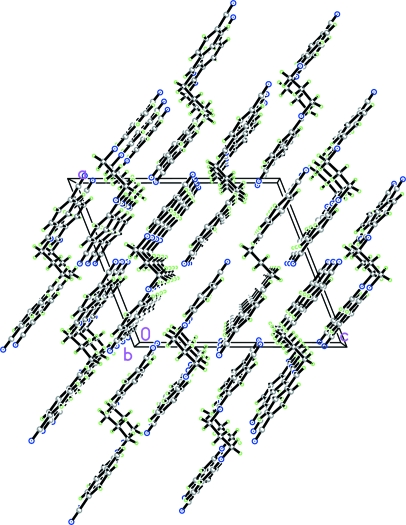
A view down the b-axis of the unit cell contents for (I), highlighting the parallel arrangement of the molecules.

## supplementary crystallographic information

### Comment

Schiff bases are one of most prevalent mixed-donor ligands in coordination chemistry. They play an important role in the development of coordination chemistry related to catalysis and enzymatic reactions, magnetism, and supramolecular architectures. Structures of Schiff bases derived from substituted benzaldehydes and closely related to the title compound, (I), are known (Li *et al.*, 2005; Bomfim *et al.*, 2005; Glidewell *et al.*, 2005, 2006; Sun *et al.*, 2004; Habibi *et al.*, 2007).

The molecule of (I), Fig. 1, has a crystallographic 2-fold symmetry. The bond lengths and angles are within normal ranges (Allen *et al.*, 1987). The group is coplanar with the aromatic ring in each half of the molecule. The planar units are parallel by symmetry, but extend in opposite directions from the central methylene bridge, the C6—C7—N1—C8 torsion angle is 178.9 (3)°. The packing of the molecules, Fig. 2, is controlled by C—H···π interactions, Table 1.

### Experimental

A solution of 1,3-propanediamine (0.1 mmol, 0.074 g) was slowly added to a solution of 4-cyanobenzaldehyde (0.2 mmol, 0.026 g) in chloroform (30 ml). Recrystallization of the resulting solid from ethanol afforded colourless crystals of (I).

### Refinement

The C9-bound H atom was located from a difference Fourier map and refined freely. The remaining H atoms were positioned geometrically with C—H = 0.93 Å (aromatic and methine) or 0.97 Å (CH_2_), and refined in the riding mode approximation with *U*_iso_(H) = 1.2*U*_eq_(C).

### Crystal data

**Table:**

C_19_H_16_N_4_	*F*_000_ = 632
*M**_r_* = 300.36	*D*_x_ = 1.263 Mg m^−^^3^
Monoclinic, *C*2/*c*	Mo *K*α radiation λ = 0.71073 Å
Hall symbol: -C 2yc	Cell parameters from 2928 reflections
*a* = 14.4982 (4) Å	θ = 3.0–30.0º
*b* = 6.9025 (2) Å	µ = 0.08 mm^−^^1^
*c* = 16.9842 (6) Å	*T* = 100.0 (1) K
β = 111.659 (4)º	Needle, colourless
*V* = 1579.67 (8) Å^3^	0.37 × 0.12 × 0.12 mm
*Z* = 4	

### Data collection

**Table:**

Bruker SMART APEXII CCD area-detector diffractometer	1544 independent reflections
Radiation source: fine-focus sealed tube	1257 reflections with *I* > 2σ(*I*)
Monochromator: graphite	*R*_int_ = 0.077
*T* = 100(1) K	θ_max_ = 26.0º
φ and ω scans	θ_min_ = 2.6º
Absorption correction: multi-scan(SADABS; Bruker, 2005)	*h* = −17→17
*T*_min_ = 0.886, *T*_max_ = 0.991	*k* = −8→8
13317 measured reflections	*l* = −20→20

### Refinement

**Table:**

Refinement on *F*^2^	Secondary atom site location: difference Fourier map
Least-squares matrix: full	Hydrogen site location: inferred from neighbouring sites
*R*[*F*^2^ > 2σ(*F*^2^)] = 0.081	H atoms treated by a mixture of independent and constrained refinement
*wR*(*F*^2^) = 0.127	*w* = 1/[σ^2^(*F*_o_^2^) + (0.0132*P*)^2^ + 5.0812*P*] where *P* = (*F*_o_^2^ + 2*F*_c_^2^)/3
*S* = 1.17	(Δ/σ)_max_ < 0.001
1544 reflections	Δρ_max_ = 0.18 e Å^−^^3^
109 parameters	Δρ_min_ = −0.37 e Å^−^^3^
Primary atom site location: structure-invariant direct methods	Extinction correction: none

### Special details

**Table:**

Experimental. The low-temperature data was collected with the Oxford Cyrosystem Cobra low-temperature attachment.
Geometry. All e.s.d.'s (except the e.s.d. in the dihedral angle between two l.s. planes) are estimated using the full covariance matrix. The cell e.s.d.'s are taken into account individually in the estimation of e.s.d.'s in distances, angles and torsion angles; correlations between e.s.d.'s in cell parameters are only used when they are defined by crystal symmetry. An approximate (isotropic) treatment of cell e.s.d.'s is used for estimating e.s.d.'s involving l.s. planes.
Refinement. Refinement of *F*^2^ against ALL reflections. The weighted *R*-factor *wR* and goodness of fit *S* are based on *F*^2^, conventional *R*-factors *R* are based on *F*, with *F* set to zero for negative *F*^2^. The threshold expression of *F*^2^ > σ(*F*^2^) is used only for calculating *R*-factors(gt) *etc*. and is not relevant to the choice of reflections for refinement. *R*-factors based on *F*^2^ are statistically about twice as large as those based on *F*, and *R*- factors based on ALL data will be even larger.

### Fractional atomic coordinates and isotropic or equivalent isotropic displacement parameters (Å^2^)

**Table:**

	*x*	*y*	*z*	*U*_iso_*/*U*_eq_	
N1	0.14554 (16)	0.8098 (3)	0.29318 (13)	0.0173 (5)	
N2	0.5053 (2)	0.1024 (4)	0.59762 (16)	0.0373 (7)	
C1	0.29164 (19)	0.6045 (4)	0.43213 (16)	0.0193 (6)	
H1A	0.2869	0.7370	0.4397	0.023*	
C2	0.3625 (2)	0.4975 (4)	0.49323 (16)	0.0211 (6)	
H2A	0.4052	0.5574	0.5422	0.025*	
C3	0.3701 (2)	0.2986 (4)	0.48155 (17)	0.0192 (6)	
C4	0.3064 (2)	0.2085 (4)	0.40896 (17)	0.0203 (6)	
H4A	0.3116	0.0761	0.4012	0.024*	
C5	0.23520 (19)	0.3172 (4)	0.34838 (17)	0.0183 (6)	
H5A	0.1921	0.2570	0.2997	0.022*	
C6	0.22689 (19)	0.5159 (4)	0.35906 (16)	0.0161 (6)	
C7	0.15182 (19)	0.6273 (4)	0.29081 (17)	0.0183 (6)	
H7A	0.1077	0.5609	0.2446	0.022*	
C8	0.06898 (19)	0.9029 (4)	0.22061 (16)	0.0185 (6)	
H8A	0.0310	0.8047	0.1811	0.022*	
H8B	0.1002	0.9855	0.1915	0.022*	
C9	0.0000	1.0233 (6)	0.2500	0.0162 (8)	
C10	0.4454 (2)	0.1869 (4)	0.54596 (18)	0.0262 (7)	
H9A	0.0367 (18)	1.110 (4)	0.2938 (15)	0.015 (7)*	

### Atomic displacement parameters (Å^2^)

**Table:**

	*U*^11^	*U*^22^	*U*^33^	*U*^12^	*U*^13^	*U*^23^
N1	0.0147 (12)	0.0211 (13)	0.0155 (11)	0.0035 (10)	0.0049 (9)	0.0024 (10)
N2	0.0446 (17)	0.0425 (17)	0.0235 (14)	0.0218 (15)	0.0109 (13)	0.0090 (13)
C1	0.0215 (15)	0.0158 (14)	0.0217 (14)	0.0001 (12)	0.0091 (12)	−0.0019 (12)
C2	0.0220 (15)	0.0242 (16)	0.0142 (13)	0.0027 (12)	0.0034 (12)	−0.0020 (12)
C3	0.0193 (14)	0.0227 (15)	0.0199 (14)	0.0046 (12)	0.0121 (12)	0.0049 (12)
C4	0.0246 (15)	0.0151 (14)	0.0268 (15)	0.0011 (12)	0.0162 (13)	0.0015 (12)
C5	0.0167 (14)	0.0191 (15)	0.0198 (14)	−0.0056 (11)	0.0075 (12)	−0.0058 (11)
C6	0.0150 (14)	0.0202 (15)	0.0165 (13)	0.0008 (11)	0.0097 (11)	0.0004 (11)
C7	0.0135 (14)	0.0229 (16)	0.0177 (14)	−0.0030 (11)	0.0048 (11)	−0.0025 (11)
C8	0.0156 (13)	0.0236 (15)	0.0159 (13)	0.0031 (12)	0.0052 (11)	0.0001 (12)
C9	0.0144 (19)	0.015 (2)	0.0174 (19)	0.000	0.0042 (16)	0.000
C10	0.0330 (17)	0.0270 (17)	0.0206 (15)	0.0072 (14)	0.0123 (14)	0.0013 (13)

### Geometric parameters (Å, °)

**Table:**

N1—C7	1.264 (3)	C4—H4A	0.9300
N1—C8	1.468 (3)	C5—C6	1.395 (4)
N2—C10	1.141 (4)	C5—H5A	0.9300
C1—C2	1.375 (4)	C6—C7	1.479 (4)
C1—C6	1.391 (4)	C7—H7A	0.9300
C1—H1A	0.9300	C8—C9	1.519 (3)
C2—C3	1.398 (4)	C8—H8A	0.9700
C2—H2A	0.9300	C8—H8B	0.9700
C3—C4	1.386 (4)	C9—C8^i^	1.519 (3)
C3—C10	1.450 (4)	C9—H9A	0.95 (3)
C4—C5	1.380 (4)		
			
C7—N1—C8	116.8 (2)	C1—C6—C5	119.1 (3)
C2—C1—C6	120.5 (3)	C1—C6—C7	122.0 (2)
C2—C1—H1A	119.7	C5—C6—C7	118.9 (2)
C6—C1—H1A	119.7	N1—C7—C6	122.3 (3)
C1—C2—C3	119.8 (3)	N1—C7—H7A	118.9
C1—C2—H2A	120.1	C6—C7—H7A	118.9
C3—C2—H2A	120.1	N1—C8—C9	110.43 (19)
C4—C3—C2	120.4 (3)	N1—C8—H8A	109.6
C4—C3—C10	120.1 (3)	C9—C8—H8A	109.6
C2—C3—C10	119.5 (3)	N1—C8—H8B	109.6
C5—C4—C3	119.3 (3)	C9—C8—H8B	109.6
C5—C4—H4A	120.4	H8A—C8—H8B	108.1
C3—C4—H4A	120.4	C8—C9—C8^i^	113.7 (3)
C4—C5—C6	121.0 (3)	C8—C9—H9A	110.8 (15)
C4—C5—H5A	119.5	C8^i^—C9—H9A	109.5 (15)
C6—C5—H5A	119.5	N2—C10—C3	178.6 (4)
			
C6—C1—C2—C3	0.5 (4)	C4—C5—C6—C7	177.7 (2)
C1—C2—C3—C4	−0.3 (4)	C8—N1—C7—C6	178.9 (2)
C1—C2—C3—C10	179.6 (3)	C1—C6—C7—N1	3.5 (4)
C2—C3—C4—C5	−0.1 (4)	C5—C6—C7—N1	−174.2 (3)
C10—C3—C4—C5	180.0 (3)	C7—N1—C8—C9	123.5 (3)
C3—C4—C5—C6	0.3 (4)	N1—C8—C9—C8^i^	−71.64 (19)
C2—C1—C6—C5	−0.4 (4)	C4—C3—C10—N2	−179 (100)
C2—C1—C6—C7	−178.0 (3)	C2—C3—C10—N2	1(14)
C4—C5—C6—C1	−0.1 (4)		

Symmetry codes: (i) −*x*, *y*, −*z*+1/2.

### Hydrogen-bond geometry (Å, °)

**Table:**

*D*—H···*A*	*D*—H	H···*A*	*D*···*A*	*D*—H···*A*
C8—H8B···Cg1^ii^	0.97	2.85	3.58	133

Symmetry codes: (ii) *x*, −*y*, *z*−1/2.
